# Radiopaque Crystalline, Non-Crystalline and Nanostructured Bioceramics

**DOI:** 10.3390/ma15217477

**Published:** 2022-10-25

**Authors:** Maziar Montazerian, Geovanna V. S. Gonçalves, Maria E. V. Barreto, Eunice P. N. Lima, Glauber R. C. Cerqueira, Julyana A. Sousa, Adrine Malek Khachatourian, Mairly K. S. Souza, Suédina M. L. Silva, Marcus V. L. Fook, Francesco Baino

**Affiliations:** 1 Northeastern Laboratory for Evaluation and Development of Biomaterials (CERTBIO), Federal University of Campina Grande, Campina Grande 58429-900, PB, Brazil; 2 Department of Materials Science and Engineering, Sharif University of Technology, Tehran 11155-1639, Iran; 3 Institute of Materials Physics and Engineering, Department of Applied Science and Technology, Politecnico di Torino, 10129 Torino, Italy

**Keywords:** biomaterials, bioactive, dentistry, bone, glass, ceramic, theranostic

## Abstract

Radiopacity is sometimes an essential characteristic of biomaterials that can help clinicians perform follow-ups during pre- and post-interventional radiological imaging. Due to their chemical composition and structure, most bioceramics are inherently radiopaque but can still be doped/mixed with radiopacifiers to increase their visualization during or after medical procedures. The radiopacifiers are frequently heavy elements of the periodic table, such as Bi, Zr, Sr, Ba, Ta, Zn, Y, etc., or their relevant compounds that can confer enhanced radiopacity. Radiopaque bioceramics are also intriguing additives for biopolymers and hybrids, which are extensively researched and developed nowadays for various biomedical setups. The present work aims to provide an overview of radiopaque bioceramics, specifically crystalline, non-crystalline (glassy), and nanostructured bioceramics designed for applications in orthopedics, dentistry, and cancer therapy. Furthermore, the modification of the chemical, physical, and biological properties of parent ceramics/biopolymers due to the addition of radiopacifiers is critically discussed. We also point out future research lacunas in this exciting field that bioceramists can explore further.

## 1. Introduction

Bioceramics have been primarily designed to treat, repair, and/or reconstruct diseased or damaged parts of the musculoskeletal system [1]. Depending on the type of interaction that they elicit in/establish with the host tissue, bioceramics can be classified as bioinert or bioactive [2]. Bioinert ceramics are passive against the environment in which they are implanted (e.g., alumina and zirconia) [3]. On the other hand, bioactive ceramics chemically interact with the surrounding tissue, causing a biological response. They show osteointegrative, osteoconductive, or osteoinductive abilities (e.g., bioactive glasses and calcium phosphates) [4,5,6,7]. Some bioceramics are also bioabsorbable, i.e., they are capable of being absorbed by the living tissues while being replaced by natural tissue; thus, their rate of dissolution and degradation is close to—and ideally coincides with—the speed of regeneration of the host tissue [2,8,9].

The application of bioceramics has proven to be effective in numerous biomedical areas, including tissue engineering [10], ophthalmology [11], otolaryngology [12], cardiology [13,14,15], orthopedics [16], dentistry [17], etc. In orthopedics, bioceramics are used to coat metallic implants [14]; as bone cement in arthroplasty, vertebroplasty, and kyphoplasty surgeries [18]; bone grafts, bone fillers [7]; and so on. [16]. In dentistry, bioceramics are used to manufacture prostheses, implants, veneers, orthodontic brackets, dental restorations, endodontic cement, etc. [19].

The world population’s life expectancy is increasing; consequently, aging is increasing the number of musculoskeletal diseases year by year. According to Global Burden of Disease (GBD) data, the prevalence of cases linked to some diseases or disorders in the musculoskeletal system was 1.71 billion in 2019. Around 436 million cases were only attributed to bone fractures [20]. As for oral disorders, including caries of deciduous and permanent teeth, chronic periodontal diseases, edentulism, and others, the number of cases was 3.48 billion [21]. These impressive numbers, and the fact that bioceramics such as those based on calcium phosphates and glasses show multiple similarities with the inorganic phase of bones and teeth [22], justify orthopedics and dentistry as the two biomedical areas with the greatest demand for these biomaterials.

**Figure 1:**
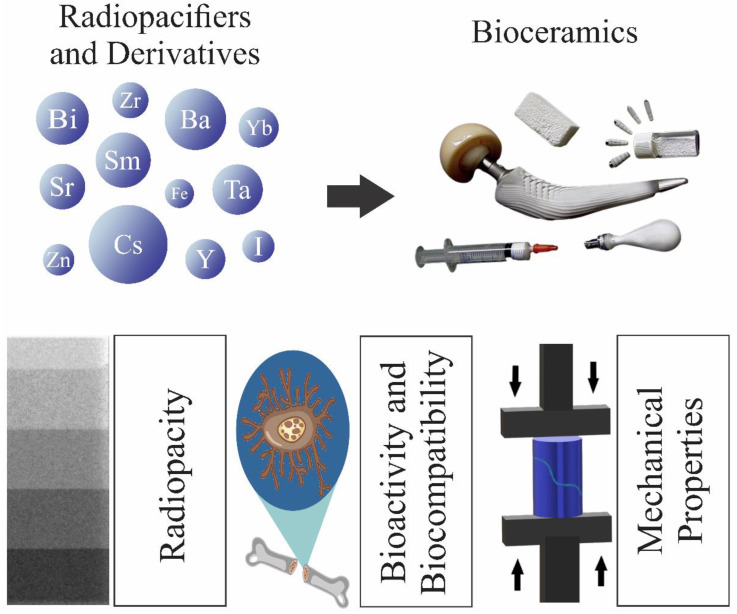
Some of the radiopacifying elements (as well as their relevant compounds) that may be added to bioceramics. Physical, mechanical, and biological properties could be affected accordingly (the image showing bone cell is from https://www.vecteezy.com/, accessed on 7 July 2022, and images indicating bioceramics are adapted from ref. [25] with permission from Elsevier).

In general, bioceramics must meet some strict requirements considering the complex environment in which they are implanted. They must be biocompatible; possess good physical, chemical, and mechanical properties; be easily processed and sterilized; and be relatively affordable and readily available [23]. However, according to the application, some more specific properties are required. For example, radiopacity is an important physical property that most bioceramics intended for orthopedics and dentistry must have. It enables the clinical evaluation of these materials at the surgical site [24]. The application of radiopaque bioceramics is indispensable in areas where radiographic visualization procedures and detecting medical devices in soft and hard tissues are challenging. Developing highly radiopaque and biocompatible bioceramics is one of the ultimate goals of bio-ceramists. Most bioceramics are intrinsically radiopaque but can still be doped/mixed with radiopacifying elements such as Bi, Zr, Sr, Ba, Ta, Zn, Y, etc., or their relevant compounds (e.g., oxides), to increase their visualization during or after medical procedures (see Figure 1). This addition might change the bioceramics’ physical, mechanical, and biological properties under investigation.

To the best of our knowledge, a comprehensive review of radiopaque bioceramics specifically addressing applications in orthopedics and dentistry is not available in the literature. Therefore, the present work aims to bridge the gap by giving an overview of this kind of radiopaque bioceramics. First, in Section 2, we succinctly review the physical phenomena responsible for radiopacity. The applications of radiopaque bioceramics in orthopedics and dentistry are summarized in Section 3. A thorough literature review of crystalline and glassy radiopaque bioceramics is provided in Section 4 and Section 5, respectively. In Section 6, polymeric–matrix composites containing bioceramics are reviewed, while Section 7 addresses nanostructured materials. Finally, we highlight the current research line and perspectives regarding future research and development (Section 8).

## 2. Principle and Physics of Radiopacity

**Figure 2:**
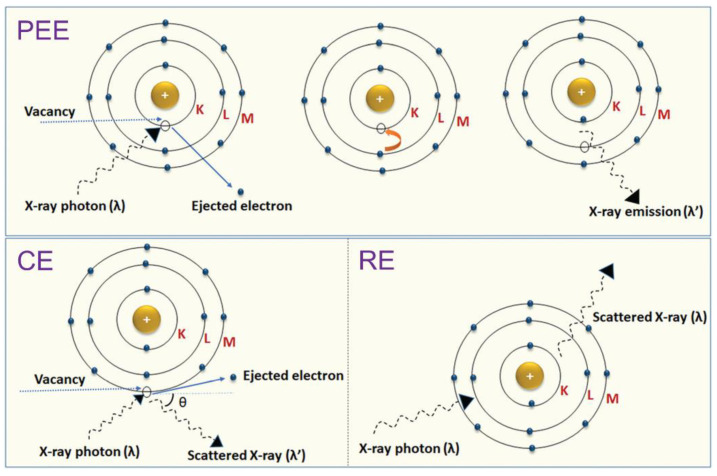
Schematics of different interactions of X-rays with matter: the photoelectric effect (PEE), Compton effect (CE), and Rayleigh effect (RE) (λ is the wavelength of the X-ray). Adapted from [33] with permission from RSC.

The characteristic of a material absorbing or scattering X-ray photons as they pass through is called attenuation. The attenuation of X-rays by a material mainly depends on electron density, the material’s thickness, and specific gravity (i.e., density). Therefore, when a material is irradiated with a parallel beam of X-ray photons, it penetrates the material and is absorbed or scattered after the interaction [26]. For example, the difference in attenuation caused by different tissues and medical devices creates a contrast between them, so greater attenuation creates higher contrast. A material is considered radiopaque if it exhibits good X-ray attenuation and produces positive contrast in the radiographic image [27]. In the diagnostic energy ranges, the photoelectric effect (PEE), Compton effect (CE), and Rayleigh effect (RE) are the three main processes by which X-ray photons can interact with the absorbing material [28]. These phenomena are briefly described herein and shown in Figure 2.

### 2.1. Photoelectric, Compton, and Rayleigh Effects

The PEE is the emission of electrons when electromagnetic radiation, such as an X-ray, hits a material. Electrons emitted in this manner are called photoelectrons. This happens when the incident radiation’s energy reaches the binding energy of the electron in the different shells of an atom (e.g., K and L layers). Subsequently, an electron at a higher energy level (such as those in M and N layers) fills the hole left by the ejected electron, and a characteristic X-ray is emitted with an energy equal to the difference between the binding energies of the two electrons (Figure 2). In general, depending on the atomic number of the atom (occupancy of different shells), photoelectrons can also be emitted from shells other than K and L (i.e., M and N). The body tissues fully absorb these low-energy characteristic X-rays. The linear attenuation coefficient of the photoelectric interaction (mPE) depends entirely on incident beam energy, tissue-effective atomic number, and tissue density. Since the mPE will be higher for materials with high atomic numbers than less dense and low-atomic-number materials, a contrast is generated between high- and low-density materials during imaging. More contrast is obtained between bone with high attenuation and soft tissue with low attenuation capacity [28,29].

In the CE, the photon is scattered by an electron with low binding energy (outer-layer electrons), which receives only part of the X-ray energy, letting it pass inside the material in another direction and with lower energy. As the energy transfer depends on the direction of the ejected electron, which is random, a photon of fixed energy can result in electrons with variable energy, with values from zero to a maximum value (Figure 2) [28,30]. The probability of Compton scattering depends primarily on the electron density of the tissues, not on the atomic number of the constituent atoms. Furthermore, it is weakly dependent on the incident energy of X-rays. At low energy X-rays, the PEE is dominant over the CE, whereas with high energy X-rays, increasing the CE reduces the image contrast [31].

The RE is a type of elastic scattering that occurs at very low photon energies (Figure 2). Since diagnostic radiology uses photons above this range, this scattering becomes important only in mammography using low photon energies. In this case, the incident photons are scattered by the atom’s electron cloud, causing slight ionization [32].

### 2.2. Radiopacity Measurement

A radiologically detectable material in the body must have sufficient radiopacity to be differentiated from the anatomical structures surrounding it. In digital and film radiography, radiopacity is measured in terms of the grayscale value, which is calculated from its digital image [34]. Each digital image consists of many pixels—the countable digital units in an image. Each pixel is related to a characteristic brightness value of the material attenuation property. In a grayscale image, since each pixel consists of 8 bits and the image has 28 bits, it reaches 256 shades of gray. Generally, the grayscale value of black and white is 0 and 256, respectively. The grayscale image of any radiodense material tends to have a gray value between 0 and 256 [35]. 

The International Standards Organization (ISO) for dental materials emphasizes that any restoration material must show radiopacity equal to or greater than pure aluminum of the same thickness as its radiopacity is very similar to dentin [36].

The gold standard and conventional customary method for radiopacity measurements is transmission densitometry. In this method, the grayscale value of photographic images (films or digitals), which is proportional to the ratio of the incident to transmitted X-ray radiation, is calculated and compared to that obtained from a standard wedge of aluminum with 10 steps from 1 cm to 10 cm.

**Figure 3:**
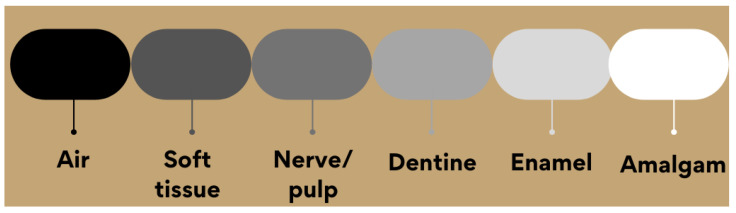
Comparison between the radiopacity of tooth constituents, air, and amalgam.

The value is expressed with respect to the equivalent aluminum thickness. As radiographic images are two-dimensional projections, that is, with no depth [37], the variation of the shades of gray between white and black represents the different anatomical characteristics of the tooth shown in Figure 3 [38,39].

For computed tomography, the quantification of radiopacity is expressed in values called Hounsfield Units (HU), in honor of the engineer Godfrey Hounsfield, the inventor of computed tomography. The HU of a material is the linear attenuation coefficient (m) normalized to that of distilled water, and at standard temperature and pressure, water and air are given a value of 0 and 1000 HU, respectively [33]. While quantitative measurements of radiopacity are possible from these imaging modalities, the extent of a material’s attenuation capability can be well-understood only from its X-ray images [38,40].

## 3. Applications: A Short Overview

### 3.1. Dentistry

Dental restorations, implants, crowns, bridges, dentures, root canal fillings, cavity liners, adhesives/cements, luting agents, and core build-up should be radiopaque in most cases. The adaptation of these materials into the anatomical structure of the mouth is analyzed by X-ray radiography to evaluate their function for long durations. Radiopaque components are usually added to these devices—some are made from bioceramics—to improve their radiopacity without compromising the mechanical properties, biocompatibility, and aesthetics [33].

The derivatives of heavy elements such as Bi, Zr, Sr, Ba, Ta, Ce, etc. are commonly used as opacifiers in dentistry. Their addition to dental ceramics as a dopant or secondary phase should not generate exaggerated radiopacity that can inhibit the dentist’s understanding of disease conditions and false-positive errors. Furthermore, the addition of excessive radiopaque fillers either compromises some properties or may cause undesirable tissue inflammations [41,42]. The names, composition, radiopacifier used in the composition, and manufacturer of some commercial radiopaque dental materials have been listed in Table 1. For example, this table shows that mineral trioxide aggregate (MTA)-based products are extensively used for dental root repair in endodontic treatments. An MTA is formulated from commercial Portland cement combined with ceramic radiopacifiers. MTAs are used to create apical plugs during apexification, repair root perforations during root canal therapy, and treat internal root resorption and pulp capping [43]. Some others are calcium silicate-based cements for endodontics. Various cements in the market provide clinicians with a wide range of options and radiopacity [44]. Some products such as AH Plus (Dentsply) and Ceramir^®^ (Doxa) are resin-modified bioceramics for endodontics and direct and indirect pulp capping. The significant release of calcium ions on moist tooth surfaces promotes the formation of hydroxyapatite (HAp) as well as tertiary dentin, leading to safe root and pulp protection. A highly alkaline pH level creates an environment that is hostile to bacteria, conducive to healing, and protective against hypersensitivity [45]. Perhaps the most widely used dental restorative composites containing bioactive glasses/glass-ceramics are glass-ionomer cements, chemically known as glass polyalkenoates [46]. They are manufactured by the reaction between fluoro-alumino-silicate glass powder (size range within 15–50 μm) and polyacrylic acid. The addition of lanthanum, strontium, or barium oxides provides radiopacity. The beneficial properties of glass ionomer cements include setting within minutes (allowing time for manipulation) and eventually forming a hard, water-resistant, bone-like substance after setting [47].

### 3.2. Ceramic Bone Cements

Calcium phosphates, calcium sulfate, magnesium phosphate, bioactive glasses, etc., are generally used in bone cements for restoration procedures. For example, they can treat osteoporotic vertebral fractures that require bone cements with radiopaque characteristics. The bone cements can confer radiopacity, but they are further doped with radiodense elements or mixed with relevant oxides. Besides the radiopacity, characteristics such as lower heat release, good shaping ability, and good mechanical and rheological properties are favorable prerequisites [55,56,57].

### 3.3. Bone Grafts and Scaffolds

**Figure 4:**
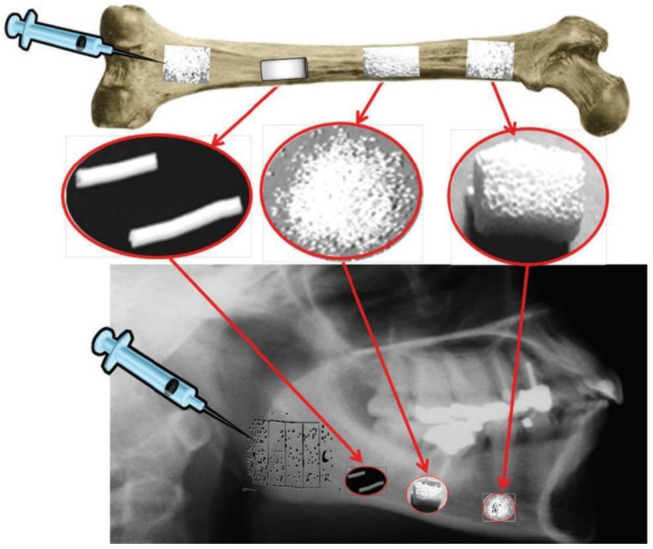
Different biomedical applications of radiopaque bioceramics in orthopedics. Reprinted from ref. [61] with permission.

With the primary goal of fixing, repairing, and regenerating bone defects, synthetic bone grafts/scaffolds are increasingly employed in modern reconstructive surgery due to the complications of using autografts or allografts. Radiopaque bone grafts, scaffolds, and implants enhance their visibility during medical imaging. Many researchers are convincingly considering the application of 3D scaffolds in regenerative medicine [58] and, specifically, bioceramics such as wollastonite, calcium phosphates, silicates, HAp, glasses, and glass-ceramics are mostly investigated to prepare scaffolds [59,60]. Figure 4 shows different applications of radiopaque bioceramics as injectable cements, grafts, fillers, and scaffolds in healing bone defects [61].

### 3.4. Composites

Polymeric composite materials are a unique class of biomaterials with important properties in engineering and biomedicine. A polymeric composite typically comprises bioceramics or other inorganic materials dispersed within a polymer matrix at micron- or nano-sizes. The radiopaque composite concept highlights the unique properties of the base polymer while improving the radiopacity of the composite device through the addition of radiopaque bioceramics [62]. Bioceramics can add additional properties to polymers generally unavailable in the polymeric matrices, such as bioactivity, osteointegration, controlled drug delivery, and many other functionalities discussed in Section 6 [63,64,65].

## 4. Radiopacifiers in Crystalline Bioceramics

There are generally two types of bioceramics: crystalline and non-crystalline (or partially-crystalline) materials. Most crystalline bioceramics are oxide or non-oxide powders that are shaped and sintered to form a solid product. Non-crystalline and partially-crystalline ceramics include glasses and glass-ceramics, respectively, that are usually synthesized through melting–casting or sol-gel routes and subsequently are subjected to controlled heat treatment or powder sintering [66,67,68,69,70,71,72,73]. Specific mechanical and biological properties should be engineered in bioceramics. The mechanical properties mainly include elasticity, hardness, compressive strength, and fracture toughness. The biological properties involve apatite-forming ability (i.e., bioactivity in osseous applications), biocompatibility, biodegradability, cytotoxicity, antibacterial properties, angiogenesis, etc. [16,74,75,76,77,78,79]. As discussed earlier, radiopacity is another essential characteristic of bioceramics that deserves to be highly considered in modern applications.

Adding some constituents with a high atomic number is the most commonly used technique to increase bioceramics’ radiopacity. This can be executed by the incorporation of substances such as bismuth oxide (Bi_2_O_3_) [80,81,82,83], zirconium dioxide (ZrO_2_) [80,82,83,84,85], strontium carbonate (SrCO_3_) [72,73,74], barium sulfate (BaSO_4_) [80,82], iron oxides (Fe_2_O_3_ e Fe_3_O_4_) [86], calcium tungstate (CaWO_4_) [81,83,87], ytterbium trifluoride (YbF_3_) [82,88,89], and titanium dioxide (TiO_2_) [82,90], among others, as listed in the Table 2. Doping bioceramics with heavy elements can also confer radiopacity. This table summarizes the radiopacity values of different bioceramics incorporated with various radiopacifying agents. In this section, we review the radiopaque bioceramics developed by adding mostly well-known radiopacfying elements and complexes based on Bi, Sr, Zr, Ba, and other elements.

### 4.1. Bismuth (Bi)

Bismuth (Bi), a metallic element with a high atomic number (Z = 83), is known for its relatively low toxicity and high stability when compared to other neighboring metals in the periodic table, such as lead (Pb), thallium (Tl), and antimony (Sb) [80,85,99]. Doping crystalline ceramics with bismuth ions increases radiopacity without significantly deteriorating mechanical properties, usually after adding radiopacifying microparticles to a ceramic matrix. Unlike ions such as barium (Ba, atomic radius (AR) = 253 pm), zirconium (Zr, AR = 216 pm), and strontium (Sr, AR = 219 pm), which are generally larger than the host lattice, the Bi (AR = 143 pm) ion does not tend to cause distortions in the crystal structure, which are responsible for changes in material properties [100]. 

**Figure 5:**
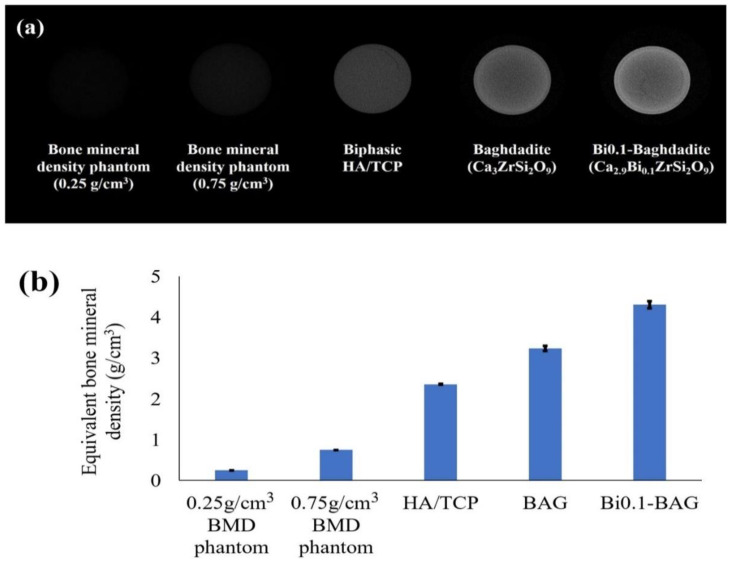
Radiopacity of baghdadite (BAG) and Bi-doped baghdadite (Bi0.1-BAG) compared to bone phantom controls and clinically used biphasic HA/TCP. (**a**) MicroCT images and (**b**) equivalent bone mineral density of disk samples investigated; from left to right: Bone mineral density (BMD) phantom = 0.25 g/cm^3^, BMD phantom = 0.75 g/cm^3^, sintered HAp/tricalcium phosphate biphasic ceramic, BAG, and Bi0.1-BAG [85].

No et al. [85] increased the radiopacity of baghdadite (Ca_3_ZrSi_2_O_9_) by 33% with the replacement of 0.1 mol of calcium with bismuth ion (Ca_2.9_Bi_0.1_ZrSi_2_O_9_). This material also revealed an improvement in radiopacity of ~115% compared to biphasic calcium phosphate (60% HAp and 40% β-tricalcium phosphate (TCP)). Figure 5 compares the radiopacities of these materials taken by microcomputed tomography (µ-CT) and measured according to MN009: bone mineral density (BMD) calibration and measurement by µ-CT using Bruker MicroCT CT-Analyzer. In this study, the bismuth-doped baghdadite shows promise as a bioceramic for orthopedic applications, with improvements in the in vitro primary human bone-derived cells (HOB) response and radiopacity compared to un-doped baghdadite. It seems that the antimicrobial properties of Bi-doped baghdadite cause enhanced HOB proliferation and activity in the presence of trace amounts of bismuth [70].

Calcium silicate cements (CSC) mixed with ~20 wt.% bismuth oxide presented radiopacity equivalent to 6.83 ± 0.48 mmAl [83]. According to Flores-Ledesma et al. [91], 10–15% of bismuth oxide must be added to an MTA to obtain the radiopacity recommended by the ISO 6876:2001 standard (3 mmAl).

In addition to radiopacity, the incorporation of bismuth ions directly influences biological and mechanical properties. Wu et al. [92] used bismuth aluminate (BiA) to confer radiopacity in calcium phosphate cement (CPC) for use in vertebroplasty. The radiopacity of CPC alone is inadequate for such an application. In this work, a 6 wt.% of BiA improved the radiopacity of CPC. The CPC samples containing less than 12 wt.% BiA showed good cell affinity. Furthermore, the CPC containing 6 and 9 wt.% BiA promoted cell proliferation and ALP activity in mouse bone marrow mesenchymal stem cells when compared to the controls. Due to their improved radiopacity and cytocompatibility, radiopaque CPCs with 6–9 wt.% BiA are expected to be a potential alternative for bone defect repair by minimally invasive surgery. In spite of an increase in stem cell proliferation of CPC in vitro with the incorporation of BiA, the compressive strength decreased. Other authors reported the same trend for other types of Bi-containing bioceramics: increasing the concentration of bismuth oxide in calcium silicate materials—such as mineral trioxide aggregate (MTA) and baghdadite—negatively affects the mechanical properties of the material [85,101]. Furthermore, it has been seen that the Bi’s presence tends to reduce the release of calcium ions [83] and causes discoloration of the tooth structure [91,102]. Moreover, Cornélio et al. [81] reported the genotoxic effect of bismuth oxide associated with white Portland cement when used at a concentration greater than 100 mg/mL. It seems that more research should still be performed to justify the mechanical and biological properties of bioceramics combined/doped with Bi derivatives.

Titanium implants coated with bismuth nanoparticles and HAp have shown a material enhancement of radiopacity and bioactivity compared to a bare implant [103].

The nanohydroxyapatite doped with a small bismuth concentration (1%) was applied onto a Ti implant surface using a supersaturated calcification solution (Bi-SCS) modified by the addition of bismuth salt. Bismuth was found to be incorporated into the apatite layer via the Ca^2+^↔Bi^3+^ substitution. The presence of Bi^3+^ ions in the Bi-SCS solution inhibits the HAp growth, thus forming nano-coatings. The results also demonstrated that the coating possesses superior antibacterial activity against Escherichia coli and Staphylococcus aureus bacteria compared to the undoped HAp coating [103].

### 4.2. Zirconium (Zr)

Zirconium has been widely used in orthopedics and orthodontics due to its chemical and physical stability as well as biocompatibility in the physiological environment [94]. Bi_2_O_3_ can be replaced with zirconium dioxide (ZrO_2_, zirconia) on some occasions, such as in dental cements, as it does not cause problems such as dentin discoloration [102]. 

Åberg et al. [93] evaluated the radiopacity of a CPC composed of β-tricalcium phosphate (β-TCP) and monocalcium phosphate monohydrate (MCPM) with different amounts of ZrO_2_. The mixture presented radiopacity of less than 3.0 mmAl for all proportions of the radiopacifier, but the cement containing 20 wt.% ZrO_2_ was found to have radiopacity greater than the commercial PMMA cement for vertebroplasty. The radiopacity increases proportionally to the concentration of ZrO2. Furthermore, the addition of zirconia to the cement increased the setting time from 20 ± 4 min to 26 ± 2 min with a 20 wt.% ZrO_2_ and reduced the compressive strength from 13.5 ± 0.6 MPa to 8.0 ± 1.2 MPa. The in vivo study demonstrated that the cement was partially resorbed and replaced by new bone, forming a bond with the host bone. The addition of ZrO_2_ was advantageous since the cement had good handling and radiopacity and, in addition, it allowed the bone to be regenerated while the cement was resorbed [93].

**Figure 6:**
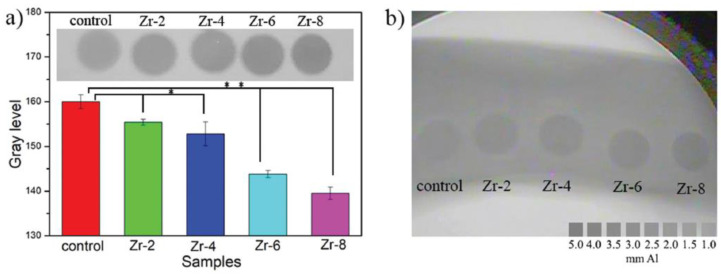
(**a**) X-ray photograph of ZrO_2_ short fiber (2–8 wt.%)-incorporated CPCs and the analyzed grey level; (**b**) X-ray photograph of cements in a bone mold. * *p* < 0.05 vs. control. ** *p* < 0.01 vs. control [94].

Zhao et al. [94] reported that adding zirconia short fibers to CPCs is a practical strategy for triggering radiopacity and improving mechanical properties. Figure 6 shows the X-ray photographs of cements with ZrO_2_ fibers. With the increase in the fiber content in the cement sample, the gray level decreases correspondingly (Figure 6a). As shown in Figure 6b, the exposure to X-ray reveals that a similar color could be detected for the bone mold and control as it is even hard to distinguish the border, while the cements with fibers are apparently darker than the bone mold [94]. In addition to the increased radiopacity, the authors reported positive effects on mechanical strength by incorporating only 2 wt.% zirconia fiber in the composition. The increase in mechanical properties due to the addition of zirconia was associated with the stress-induced transformation of tetragonal ZrO_2_ to the monoclinic phase, accompanied by a volumetric expansion of approximately 4 to 5% that can stop propagating cracks and improve mechanical performance [104]. At high concentrations (above 20 wt.%), the crack formation during the synthesis that occurred due to a mismatch of thermal and physical properties between ZrO_2_ and the matrix that degraded the mechanical properties. This can also explain the decrease in the mechanical strength of the CPC with the addition of up to 40 wt.% of ZrO_2_ developed by Alberg et al. [93] and the increase in this property for the CPC incorporated with only 2 wt.% ZrO_2_ short fibers. Cell proliferation was also evaluated for CPC with up to 8 wt.% ZrO_2_ short fibers [94]. The results were more promising than the control, as the surface was practically fully covered by cells, indicating the ability of ZrO_2_ to promote cell growth on the surface of the biomaterial, eventually facilitating osseointegration [94]. 

Chen et al. [95] attempted to reduce the cytotoxic effects associated with the presence of Bi in MTA with the partial replacement of Zr. In this study, Bi_2_O_3_ powders were replaced with ZrO_2_-doped Bi_2_O_3_ powders by adding zirconium oxide through precipitation processes. They obtained a powder with a Bi_2-x_Zr_x_O_3+x/2_ stoichiometry. The research showed an increase in radiopacity from 4.69 ± 0.23 to 5.57 ± 0.28 mmAl with the replacement of only x = 0.2 mol of Bi with Zr. The powders developed at higher synthesis temperatures were more radiopaque.

### 4.3. Strontium (Sr)

Strontium is a trace element in the human body [105]. It belongs to group II on the periodic table, like calcium; in fact, they respond similarly in the body. Among the bioactive metals, strontium ions (Sr^2+^) offer the best responses to the body in terms of bone regeneration [106]. Strontium’s application as an additive in bone cements is recommended because it is a therapeutic component for treating osteoporosis [96], in addition to absorbing greater amounts of X-rays than Ca, making the material more radiopaque [24,105].

Researchers have reported an increased radiopacity of ceramic materials through the replacement of certain constituents by strontium-based compounds. Schumacher et al. [97] verified that the replacement of 1.10 and 2.21% of calcium carbonate (CaCO_3_) by strontium carbonate (SrCO_3_) is effective in terms of increasing the radiopacity of a tricalcium phosphate-based cement. You et al. [107] used Sr to dope tricalcium silicate (Ca_3_SiO_5_)—a component of MTA—and compared its radiopacity with a cement containing 10 wt.% of Bi_2_O_3_, verifying better radiopacity for the one containing Sr. Replacing magnesium ions (Mg) by Sr in magnesium phosphates also showed promising results. The radiopacity of struvite (MgNH_4_PO_4_.6H_2_O) was increased by 1.9–3.1 times over its pure composition by adding 8.2 to 24.6 mol% of a Sr dopant [96]. Recently, Souza et al., developed a new cement composed of calcium aluminate, strontium aluminate powders, and chitosan/glycerin solution. The cement properties were optimized through a 2^k^ factorial experimental design. Their model suggested an optimized composition for possible application as bone cement with an average T_max_ of 40.34 °C, a compressive strength of 7.75 MPa, and radiopacity of 3.76 mmAl, all above the standard requirements. Good radiopacity was obtained due to the utilization of strontium aluminate [108].

In addition to its potential as a radiopacifier, strontium influences biological, chemical, and physical properties. Sr^2+^ reduces the osteoclastogenic activity and apoptosis of mature osteoclasts while promoting osteoblast proliferation and differentiation, stimulating the formation of new bone [96]. The substitution of Ca ions with Sr ions in calcium phosphates, such as HAp, can ideally vary from 0 to 100%. As this range increases, the phosphate solubility also increases [21,105] due to the expansion in the crystal structure [97] caused by larger Sr ions [100]. 

The replacement of 1.10 and 2.21 wt.% of CaCO_3_ precursor with SrCO_3_ in α-tricalcium phosphate cement improved its mechanical strength and controlled the release of Sr ions [97]. On the other hand, the replacement of 8.2, 16.4, and 24.6% of Mg^2+^ with Sr^2+^ resulted in the fragility of the struvite-based scaffold due to the excessive increase in porosity; however, it made the material more degradable and provided osteogenic properties superior to magnesium phosphate [96]. Additionally, doping Ca_3_SiO_5_ with 10% of Sr did not significantly affect hardness after 7 days of cement hydration [85], indicating that the replacement with Sr did not considerably change (at least) the hardness of this cement.

### 4.4. Barium (Ba)

Barium sulfate is routinely used in gastroenterology as a contrast medium [98] and is among one of the most researched effective radiopacifiers, together with zirconium oxide (ZrO_2_) and bismuth oxide (Bi_2_O_3_), to improve the visualization of non-radiopaque materials due to its high atomic number (Z = 56) [92,100,109]. It is a white element and, therefore, should not cause color changes in dentistry [91]. 

Despite being one of the main radiopacifying agents in polymeric materials, barium sulfate and Ba dopants have not been shown to be effective in increasing the radiopacity of ceramic cements when added to their compositions in a proportion of up to 20 wt.%. Therefore, to achieve the recommendations set out in the regulations (>3 mm of Al), higher concentrations of Ba are required [80,85]. Nevertheless, this higher concentration does not negatively affect the material’s mechanical properties, which tend to increase or remain the same after adding some amounts of Ba. Myat-Htun et al. [110] explained that introducing barium (Ba^2+^) ions in akermanite (Ca_2_MgSi_2_O_7_)—a magnesium calcium silicate—will improve the densification of the ceramic and, consequently, its mechanical properties. They also found that the doped material was wholly covered with apatite crystals after 21 days of immersion in a simulated body fluid solution (SBF). This indicates that Ba^2+^ promoted the deposition of apatite crystals.

Due to the need to use high concentrations of Ba for augmenting radiopacity in ceramics, some studies have reported that BaSO_4_ presumably has toxic effects, is not biodegradable, is not biocompatible, and could generate severe rejection reactions in the surrounding tissues [111,112]. However, the effects of Ba are still under debate; in contrast, for example, Liu et al. [113] reported that the BaSO_4_ could increase the mechanical behavior and radiopacity while not suppressing the good biocompatibility, biodegradability, and osseointegration of injectable CPC mixed with starch. In addition, the replacement of calcium ions with barium ions has aroused interest in researchers due to their participation in bone repair and regeneration (osteogenesis) [110]. Alshemary et al. [114] reported that Ba ions stimulate the in vitro formation of HAp in calcium phosphate bioceramics.

### 4.5. Other Elements

Achieving a level of radiopacity sufficient to distinguish the biomaterial and the surrounding tissue is a difficult task, mainly due to the limited number of dopants/additives capable of being used [80]. Some authors have investigated other radiopacifiers in order to evaluate their impacts on bioceramics.

The addition of iron oxide nanoparticles to HAp was explored by Ajeesh et al. [86]. They sintered co-precipitated HAp and Fe_3_O_4_ powders at 1200 °C and obtained a Fe_2_O_3_–HAp composite ceramic. The authors reported an increase of up to 38% in opacity with 60 wt.% of iron oxide in the composition. However, the level of radiopacity decreased by reducing the element to non-toxic levels (less than 40 wt.%). Furthermore, HAp maintained its phase identity for all composites, increasing cell viability and providing good adhesion.

An alternative to replace MTA—which mainly contains bismuth oxide as a radiopacifier—was studied by Costa et al. [89]. The researchers produced a calcium silicate ceramic with 30 wt.% YbF_3_ (CSC/YbF_3_). The ceramic presented radiopacity similar to that of MTA, ~5 mmAl, without altering the physicochemical and biological properties of calcium silicate. Furthermore, the material showed significantly higher mechanical strength than MTA, that is, 39.46 ± 5.78 MPa after 24 h, while MTA showed a strength of 16.1 ± 3.95 MPa after the same period, reaching 59.64 ± 14.60 MPa and MTA 32.01 ± 7.76 MPa after 21 days. Other properties reported included the absence of cytotoxic effects, low solubility, and bioactivity.

Bosso-Martelo et al. [83] added different radiopacifying agents, including calcium tungstate (CaWO_4_) (30 wt.%), in CSCs and compared them with MTA cement. According to their results, cement with CaWO_4_ had a closer solubility to MTA, with a radiopacity of 5.67 ± 0.5 mmAl—close to ZrO^2−^ and Bi_2_O_3_-added CSCs—and a shorter setting time. A more recent study replaced some of the calcium silicates with CaWO_4_ in ceramic cements, reaching radiopacities higher than the ISO recommendation (3 mmAl). This replacement resulted in a change in the cement structure and a consequent decrease in the release of Ca ions with the increase in CaWO_4_, a probable reason for the decline in cell viability and proliferation. The 10% replacement increased radiopacity without adversely affecting mechanical properties and the ability to proliferate and differentiate cells [87].

## 5. Radiopacifiers in Glasses and Glass-Ceramics

Bioactive glasses (BGs) and glass-ceramics (BGCs) belong to the third generation of biomaterials that, once implanted, can help the body heal itself [115]. BGs were first introduced in 1969 by Larry L. Hench in the USA [116]. They offered great versatility for bone and tissue engineering/regeneration [117]. Furthermore, through a controlled heat treatment of bioactive glasses, an internally nucleated monolithic sample or a sintered/partially-crystallized glass powder compact, called glass-ceramic, was made [118,119,120]. BGCs are, in principle, tougher and stronger than BGs. BGs are developed by melting–quenching or sol-gel methods, and in some cases, BGCs can also be obtained without the need for any post-synthesis thermal treatment. Sol-gel glasses are promising in developing modern bioactive glasses, i.e., nanoporous powders and even small monoliths [121]. They offer higher purity and homogeneity than melt-derived glasses and exhibit faster bioactivity over a broader compositional range due to their high surface areas [122,123,124,125]. Mesoporous bioactive glasses (MBGs) are the latest generation of BGs developed by Yan et al. [126] through the application of the surfactant-induced self-assembly of the inorganic constituent of bioactive glasses. MBGs are mesostructured materials with a well-ordered pore arrangement, a high surface area, and an average porosity in the range of 2 to 50 µm. They are highly reactive and show drug-delivery ability.

Since their development, BGs’ composition has been modified by numerous elements, including radiopacifying agents. For instance, bioactive glasses containing Sr, Bi, Zr, Zn, Ba, etc., have been tested, and some of these materials have unique properties for use in medicine and dentistry. They are described and discussed in this section.

### 5.1. Strontium (Sr)

Many BGs and BGCs that host unique structural modifiers such as strontium (Sr) have been studied, considering that Sr is a radiopacifier and plays a vital role in the human body. For example, in some cases, Sr can replace Ca in the physiological pathway or be deposited in the bone mineral structure [127,128,129].

The presence of Sr in MBGs and BGs shows promise due to its high radiopacity and degradation rate. High levels of radiopacity can also be achieved by incorporating very high levels of strontium (equivalent to 40 mol% SrO) in the glass composition. Studies show that considerable amounts of Sr can also decrease the dissolution rate of the material [130,131,132].

O’Brien et al. [130] argue that the Sr^2+^ ion has a dual mode of action, i.e., increasing bone formation by osteoblasts while simultaneously decreasing bone resorption by osteoclasts, which makes it very suitable in the treatment of osteoporosis. These results support the ability of Sr to accelerate bone reconstruction, as explained by Draghici et al. [133] and Maciel et al. [134].

Zhao et al. [135] developed Sr-doped, MBG-based scaffolds (composition: 57.2SiO_2_–7.5P_2_O_5_–35.3(Sr+CaO) wt.%) by using a 3D printer and observed the material’s ability to form apatite in vitro, stimulate the proliferation and differentiation of osteoblastic cell lineages, and promote angiogenesis. Computed tomography images revealed the high radiopacity of these scaffolds after 8 weeks of implantation in the cranial region of rats. Zhang et al. [136] worked on another 75SiO_2_–15CaO–5P_2_O_5_–5SrO (mol%) MBG-based scaffold for periodontal regeneration implanted in rats showing osteoporosis. In the images provided by computed tomography, it was possible to attest the radiopacity of the MBG with Sr along with the regeneration of the rats’ trabecular bone. The results indicated a 46.67% new bone formation after using Sr-MBG, while the Sr-free MBG scaffolds and control samples showed 39.33% and 17.50%, respectively.

In addition to bioactive materials, Höland et al. [137] at Ivoclar Co. could develop a series of radiopaque Sr-doped fluoroapatite glass-ceramics as inert dental prostheses. Dental glass-ceramics of this kind are developed by the controlled crystallization of oxide glasses and form an important group of biomaterials used in modern dentistry. They are designed to have exceptional aesthetics, translucency, high strength, chemical durability, wear resistance, biocompatibility, low thermal conductivity, hardness, and radiopacity [138]. Höland et al. [137] precipitated Sr-doped fluoroapatite in SiO_2_–Al_2_O_3_–Y_2_O_3_–SrO–Na_2_O–K_2_O/Rb_2_O/Cs_2_O–P_2_O_5_–F base glass compositions. The crystal phase formation, main thermal properties, optical properties, and radiopacity were compared with a reference Ca-fluoroapatite glass-ceramic. The glass-ceramics contained: Sr-fluoroapatite (Sr_5_(PO_4_)_3_F), leucite (KAlSi_2_O_6_), nano-sized NaSrPO_4_, pollucite (CsAlSi_2_O_6_), and Rb-leucite (RbAlSi_2_O_6_) depending on the composition and heat treatment schedule used. The Sr-fluoroapatite was internally precipitated in the glassy matrix, demonstrating a needle-like morphology, while the formation of leucite, pollucite, and Rb-leucite was based on a surface crystallization mechanism. The authors could increase the radiopacity by developing the Sr-fluoroapatite and leucite glass-ceramic for use as dental veneers. However, the highest increase in radiopacity was observed for the Sr-fluoroapatite-pollucite type glass-ceramics, which showed a five-fold increase in radiopacity!

### 5.2. Bismuth (Bi)

Some studies address the influence of bismuth (Bi) in the area of glassy materials due to its ecologically friendly characteristics, replacing other metallic elements with high toxicity such as lead [139,140]. In addition to this ecological aspect, Bi-doped glasses or glass-ceramics are known for their high refractive index, low photon energy, and low glass transition temperature. These materials also stand out for the high radiopacity conferred by bismuth and are desirable in optical and electronic devices as well as thermal and mechanical sensors, among other applications [141,142].

Bi has emerged as a new element to be included in MBG and glass-ceramics due to its favorable properties such as biocompatibility and low toxicity. In addition, Bi’s inclusion can increase these materials’ mechanical, biological, and osteogenesis properties. The radiopacity of the materials with the presence of Bi improves the image contrast of the treated area by X-ray radiography and computed tomography [92,143].

**Figure 7:**
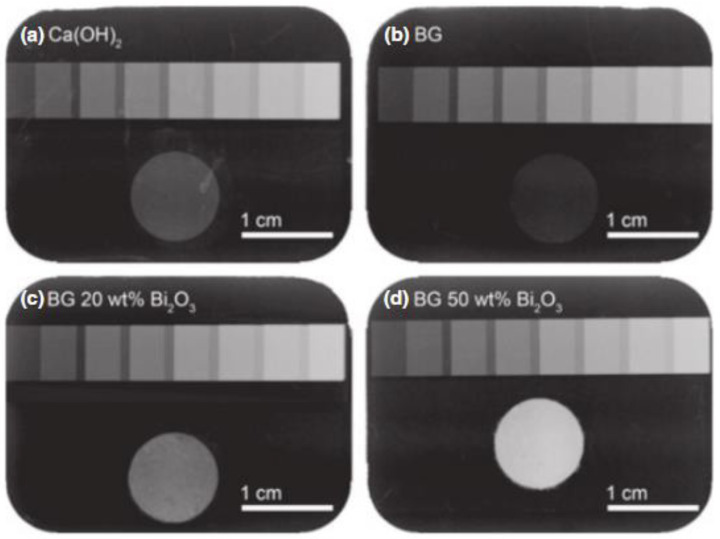
Radiography image of melt-derived BGs and aluminum-scale reference material: (**a**) calcium hydroxide, (**b**) 45S5 Bioglass^®^, (**c**) 45S5 Bioglass^®^ with 20 wt.% of Bi_2_O_3_, and (**d**) BG with 50 wt.% of Bi_2_O_3_ [145].

Heid et al. [144] observed that Bi-doped 45S5 glass maintained a higher radiopacity and induced a quicker pH increase when compared to the glass composition without Bi. Mohn et al. [145] reported that BG particles with up to 50 wt.% of bismuth oxide showed radiopacity equivalent to 4.94 mm of aluminum, as shown in Figure 7. The introduction of bismuth in the 45S5 composition altered the alkaline dissolution rate and bioactivity in vitro only for high amounts of bismuth oxide.

### 5.3. Zirconium (Zr)

**Figure 8:**
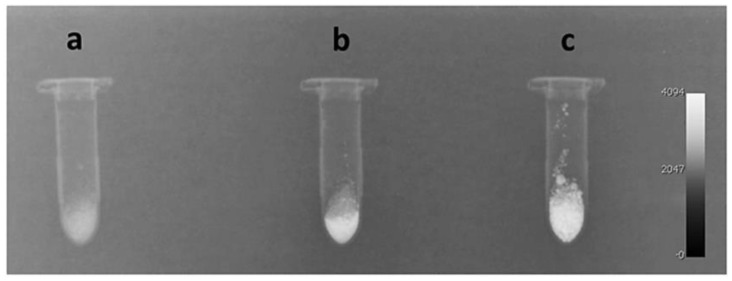
X-ray image showing the radiopacity comparison of a SiO_2_–CaO–P_2_O_5_–ZrO_2_ gel-glass powder: (**a**) without Zr; (**b**) doped with 7 wt.% Zr; (**c**) doped with 15 wt.% Zr [146].

Zr-containing BGs and BGCs have constituted an active field of research for over 20 years. This element can be added to glasses or ceramics for at least three distinct purposes: (1) to develop radiopaque bioactive powders and mix such powders with bone cements or bone fillers to improve contrast during a radiological follow-up, (2) to develop inert and durable glass-ceramics for dental restoration, and (3) to reinforce BGs or BGCs. For example, the use of SiO_2_-ZrO_2_ glasses as adjuvant radiopacity fillers has been suggested for light-cured dental composites, bone graft substitutes, and bone cement, among others, as illustrated in Figure 8 [146,147].

During the evaluation of the bioactivity of glasses and glass-ceramics doped with ZrO_2_, Montazerian et al. [147] observed an increase in the proliferation of osteoblastic MG-63 cells. The addition of Zr to the glass’ structure delayed the kinetics of bioactivity, i.e., a longer period (72 h) was required for the formation of carbonated HAp in comparison to the samples without Zr (24 h). Interestingly, however, HAp formation occurred faster on the surface of the glass-ceramics with Zr than on the glass of the same composition because the glass depleted from Zr ions as ZrO2 crystals formed in the glass-ceramics.

In addition, in the biological environment, glasses containing Zr have high chemical durability as a reflection of the interaction between Zr ions in the glass structure. Zr acts as an intermediate glass former that can link the silicon tetrahedron, making the glass more stable [147,148]. This effect can be observed through the smaller pH variation for the glass doped with Zr, indicating that the solubility of the glasses decreases with the increase in the ZrO_2_ concentration [146]. According to Yin et al. [149], this is beneficial and desirable for cell adhesion and growth. Nevertheless, reducing surface reactivity can decrease the material’s bioactivity.

### 5.4. Barium (Ba)

Barium (Ba) has a high intrinsic radiopacity, very close to Sr. This element is incorporated as compounds (e.g., oxides or carbonates) during glass preparation, thereby imparting radiopacity and modifying other properties, including mechanical and optical characteristics [132,150,151].

Barium and barium compounds are valuable candidates for medical applications due to their unique properties, such as their high density, high polarizability, and radiopacity. As briefly mentioned, the radiopacity of barium sulfate and other barium-containing compounds enables the detection of body vessels and implants [152]. This feature helps radiologists determine the orientation and status of body ducts (e.g., angiography to examine cardiovascular channels and barium swallow studies to examine the gastrointestinal tract). Meanwhile, this feature helps the orthopedic surgeon to place the implant in the correct position and subsequently inspect the condition of the implant [90,153].

**Figure 9:**
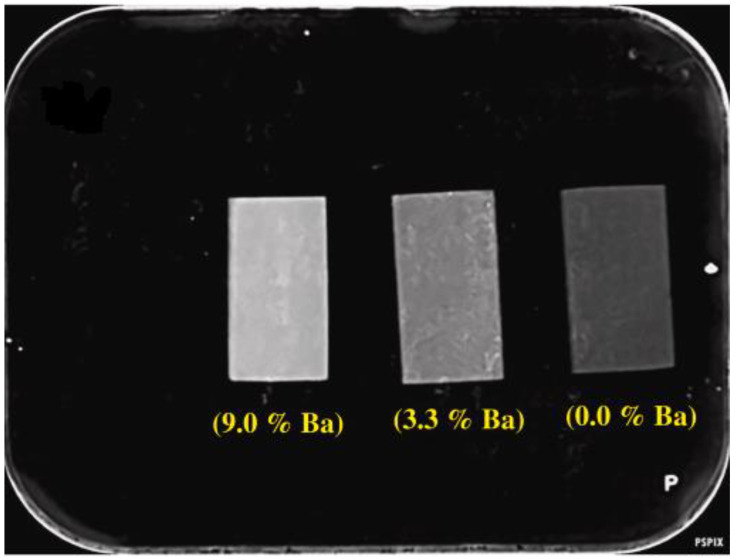
Radiograph of SiO_2_–CaO–P_2_O_5_–B_2_O_3_–Na_2_O–Al_2_O_3_ glass samples with 0% BaO, 3.3% BaO and 9% BaO (in mol%) [154].

Khoeini et al. [154] investigated BGs containing barium oxide. They observed that the replacement of calcium with barium enhanced radiopacity. Furthermore, they reported a uniform opacity of the sample, as shown in Figure 9.

The amount of the radiopaque element must be considered an important variable. For example, the radiopacities of the 35 to 40% Ba-containing glass samples exceed that of the tooth enamel, demonstrating that it is possible to formulate highly radiopaque composites by adjusting the amount of Ba in the glassy component. Nevertheless, large concentrations of Ba in composite bone cements with BGs can delay setting time, accelerate water degradation, or form secondary particles within the base material [150].

### 5.5. Magnesium (Mg)

Magnesium (Mg) is an important mineral of the bone matrix that is contained in enamel, dentin, and bone structure. Magnesium oxide (MgO) has also been considered a substitute for CaO in BG and BGC compositions to modify their biological characteristics and mechanical properties [129].

Tamura et al. [155] reported that 44.7CaO–34SiO_2_–16.2P_2_O_5_–0.5CaF_2_–4.6MgO (wt.%) glass and the derived glass-ceramics exhibited a reactive radiopaque layer, where new bone formation appeared within two weeks. The authors also reported a decrease in the degradation rate of the glass-ceramic samples as compared to glass powder due to the presence of crystalline phases (apatite and wollastonite) that increased the chemical stability of the materials. This is the likely reason for the higher flexural strength (180 MPa) that glass-ceramics exhibited compared to the glass with a similar composition (72 MPa).

**Figure 10:**
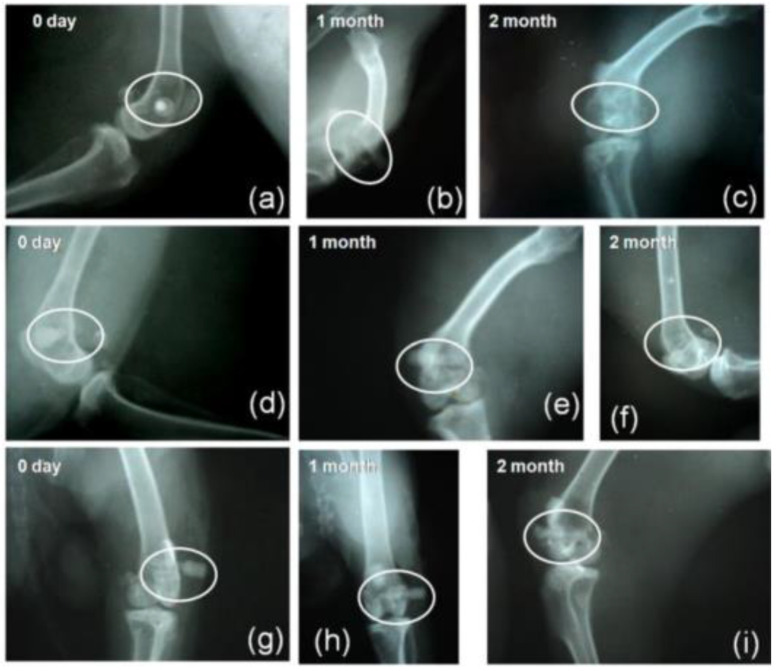
Radiographs of BM (**a**–**c**), BMH (**d**–**f**) and BMG (**g**–**i**) placed in bone immediately after implantation (day 0) and 1 month and 2 months after surgery [156].

Mahato et al. [156] compared a magnesium-based metallic implant (Mg-Zn-Ca) (BM) with and without HAp (BMH) and bioactive glass S53P4 (BMG) coatings. Figure 10 shows the radiograph of the samples in a femoral bone defect in mice. It is possible to observe that the BM degrades after 2 months of surgery but appears as a radiodense material on the day of implantation (day 0). The BM implant was radiopaque over the 2 months of implantation despite a distinct radiolucent gap between the bone and the implant (Figure 10). It is noticeable that the BMG-coated implant presented the highest radiopaque density among the other implants (Figure 10). In addition to improved radiopacity, the authors reported a superior corrosion resistance in vitro, evaluated by the lowest I_corr_ (68 µA) and E_corr_ (−296 mV) values compared to BMH samples (I_corr_ = 297 µA; E_corr_ = −1420 mV). In addition, the enhancement of the apatite precipitation capacity on the Mg alloy samples was observed, corroborating the bone formation verified by the biological tests. 

### 5.6. Zinc (Zn)

Zinc-doped glass-ceramics and glasses have been shown to stimulate wound healing by increasing osteoblast differentiation and osteoblast DNA content. Zn-doped biomaterials have also shown antibacterial efficacy by killing many bacterial strains commonly associated with infection after orthopedic surgery [130]. However, Sharifianjazi, Moradi [129], emphasized the importance of controlling the Zn content, as the glass transition temperatures can be reduced with the increase in Zn and can also deteriorate the bioactivity of glass or glass-ceramics.

For transarterial embolization (TAE) procedures, zinc-silicate glasses are promising due to their intrinsic radiopacity. Hasan et al. [157] produced microspheres of a multi-component system (0.562SiO_2_–0.035CaO–0.188ZnO–0.068La_3_O_2_–0.042TiO_2_–0.035MgO–0.035SrO–0.035Na_2_O, mol%), which was radiopaque, biocompatible, and non-degradable, as required for the treatment and monitoring of the emboli. Kehoe et al. [158] synthesized the same glass and emphasized the increase in the radiopacity values evaluated by a CT analysis, which was induced by the modifier ions such as zinc. In another work, the authors registered an increment from 3223 to 12,042 HU for this property of the glass [159].

### 5.7. Yttrium (Y)

**Figure 11:**
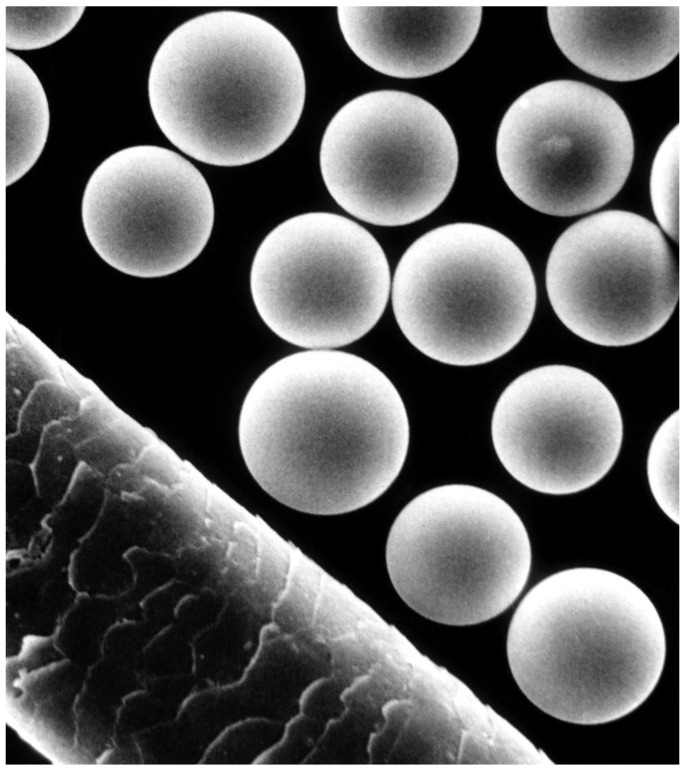
Radiopaque ^90^Y glass microspheres (TheraSphere^®^) next to a human hair. Adapted from [169].

In 1999, ^90^Y-containing inherently radiopaque glass alumino-silicate microspheres (Figure 11), after being approved by the Food and Drug Administration (FDA), were commercialized under the TheraSphere^®^ brand [160]. The radioisotope ^90^Y emits short-range β radiation with a short half-life (64 h), thereby killing cancer cells. They are used to treat patients with primary liver cancer that cannot be surgically removed (unresectable hepatocellular carcinoma) [161,162]. This product is used in more than 200 specialized centers worldwide. After being injected into the hepatic artery, microspheres containing the radioactive ^90^Y isotope can be deposited in the capillary bed of the liver to induce radioembolization effects, thereby killing cancer cells and decreasing the blood flow to the malignant tumor with an observed significant reduction of the tumor mass; as a result, other follow-up therapies, such as surgery or transplants, can then be performed [163]. In addition, the life expectancy for terminally ill patients has increased from 5–7 months to 12–24 months. Compared to other types of cancer treatments, such as chemotherapy, TheraSphere^®^ has fewer side effects and only causes flu-like symptoms such as fatigue, mild fever, or abdominal pain that persists for several days in some patients after treatment [163]. In 2006, Bretcanu and Evans published a comprehensive review of the clinical applications of TheraSphere^®^ for the liver cancer therapy [164]. More recently, Daniel Boyd’s team at Dalhousie University, Canada, has developed a new radioactive glass that activates radioembolization and shows potential in treating cancer. This material, trademarked as Eye90 Microsphere™ glass, is being commercialized by ABK Biomedical Co. [165,166,167]. To learn more about radiopaque BGs for targeting cancer, interested readers can refer to a recent review by Moeini et al. [168].

### 5.8. Other Elements

Other elements can be added to BGs and BGCs to trigger radiopacity. For example, Dubok et al. [170] pointed out the use of iodine (Z = 53) and cesium (Z = 55) to replace the hydroxyl and sodium groups in the glass structure, respectively. Some aspects, such as the instability and volatility of the element, should be considered for the utilization of cesium. Additionally, the authors discussed the use of divalent lanthanide elements to increase the radiopacity of calcium phosphates in glass-ceramics. Ytterbium (Z = 70) can improve the radiopacity of glass-ceramics with only 1 wt.%. Calcium’s replacement with samarium (Z = 62) is recommended for the same functionality [148].

Bauer et al. [171] reported an interesting approach to adding niobium to vitreous and polycrystalline phosphate glasses/glass-ceramics. According to the authors, this element is intriguing because it enhances the chemical durability of dental adhesives and improves their biocompatibility, mechanical properties, and radiopacity.

Furthermore, Alhalawani et al. [172] fabricated a poly(acrylic) acid cement with SiO_2_–CaO–P_2_O_5_–ZnO–SrO glass-ceramics containing 0.002 and 0.005 mol.% Ta_2_O_5_ (Ta1 and Ta2, respectively). All the cements exhibited a radiopacity higher than that of the aluminum scale (290% and 300% higher than Al for Ta1 and Ta2, respectively). Grishchenko et al. [173] showed that samples of 45S5 Bioglass^®^ containing at least 4 wt.% of Ta_2_O_5_ were bioactive and radiopaque. Adding between 4 and 10 wt.% of tantalum oxide in the glass-ceramic structure improved the mechanical properties and made it possible to monitor bone tissue regeneration. However, an 18 wt.% of Ta_2_O_5_ suppressed the bioactivity. For this condition, the apatite layer was not observed even after incubation for 30 days in SBF solution. The suggested reason for this result was the decrease in the calcium concentration in the glass—and hence the very low calcium amount released in the SBF—through the formation of insoluble crystals of CaTa_2_O_6_.

## 6. Polymer-Based Composites/Hybrids

The application of different composite/hybrid materials in medicine and dentistry has been growing. One interdisciplinary approach that has been investigated for a long time is the treatment of various types of bone- and tooth-related diseases and disorders by utilizing biopolymer–ceramic composites or organic–inorganic hybrids, which combine the properties of two materials and achieve enhanced biological and biomechanical properties along with radiopacity. They show appropriate properties for applications that require strength, durability, and biocompatibility. Recently, a wide range of biopolymers, such as poly(L-lactic acid), poly(L-lactide-co-glycolide), poly(methyl methacrylate) (PMMA), poly(ε-caprolactone), etc., have been studied for different biomedical procedures because of their good biodegradability and biocompatibility. They can be processed by advanced additive manufacturing techniques for the fabrication of custom-made prostheses. Radiopaque bioceramics or heavy inorganic elements can contribute significantly to the medical success and evaluation of these biomaterials as they improve radiopacity and biocompatibility similar to the degrees found in natural bone and teeth [174,175,176,177]. Most of the bioceramics discussed in the previous sections can infer radiopacity from the polymeric matrix composites. In addition, the heavy inorganic elements mentioned above can contribute at a molecular scale to the formation of radiopaque polymeric hybrids. Table 3 collects some recent examples, with interesting results and stimulating challenges ahead. However, many exciting opportunities still deserve to be explored by researchers [178,179,180].

**Figure 12:**
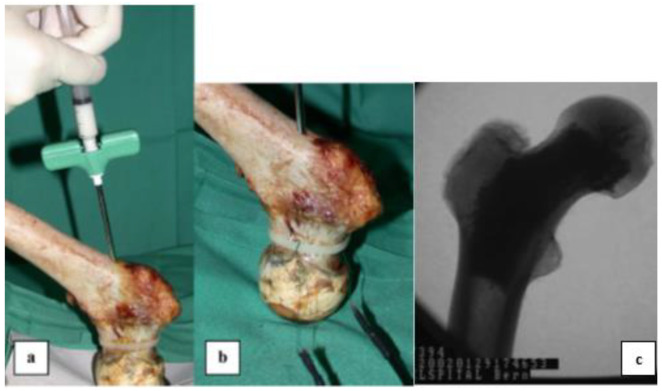
(**a**) Injection of Cortoss^®^ composite bone cement. (**b**) Femur with attached temperature sensor. (**c**) Radiological appearance of augmented femora [187].

Table 3 shows that, for example, the polymeric cements (mostly PMMA-based) that have been used in kyphoplasty, vertebroplasty, femoroplasty, and arthroplasty for a long time can fix the bone–implant interfaces and treat fractures as well. The radiopacity of such materials can be increased by adding contrast dyes or ceramic second phases (Table 3), conventionally micron-sized and inert bioceramics such as barium sulfate and zirconium dioxide [181,182]. Many examples of such cements have been studied and reviewed in [183,184,185,186]. For instance, an acrylic-based (BIS-GMA) cement in conjunction with bioactive ceramic is highly radiopaque and has good mechanical properties. One such cement, Cortoss^®^, is currently used in clinical applications for vertebroplasty and is a potentially valuable alternative to PMMA (Simplex^®^ and Spineplex^®^). The bioactive ceramic (combeite; diameter of ~5–30 μm) component in Cortoss^®^ has been proven to facilitate bone growth directly on the implant. Such a composite can also be used in femoroplasty, a prophylactic cement augmentation of the proximal femur that may reduce fracture risk. Figure 12 shows the examination of Cortoss^®^ in nine pairs of osteoporotic human cadaveric femora (seven males and two females, mean body weight 72 kg, and mean height 169 cm). The good radiological appearance of augmented femora, which is evident in Figure 12c, is mainly due to the addition of inorganic glass-ceramic filler [187,188,189].

**Table 3:** mmAl radiopacity of different dental and orthopedic materials incorporated with various radiopaque bioceramics.

Filler	Base Polymer	Filler Concentration (wt.%)	Application	Radiopacity (mmAl)	Ref.
Ta_2_O_5_	Bisphenol-A-glycidyl methacrylate (Bis-GMA), trimethylene glycol dimethacrylate (TEGDMA), 2-hydroxyethyl methacrylate (HEMA)	1–10	Dental adhesive	<1 mm	[190]
Fiber glass and zirconia	Ethoxylated bisphenol-A-dimethacrylate (bis-EMA), TEGDMA, diurethane dimethacrylate (UDMA)	0–25	Dental Composite resin	4.6	[191]
Sr-doped HAp	Bis-GMA, HEMA	10	Dental adhesive	1.1	[106]
Bi_2_O_3_, S_i_O_2_, YbF_3_	HEMA, UDMA, TEGDMA, Bis-GMA, glycerol dimethacrylate (GDMA)	10	Dental adhesives	>1 mm	[192]
CeO_2_	TEGDMA, Bis-GMA, HEMA	0.36–5.76 vol%	Dental adhesives	>1 mm	[193]
CaWO, YbF_3_, BaSO_4_	Bisphenol-A	20–120	Dental root canal sealer	2.6	[194]
Nb_2_O_5_	Bis-GMA, HEMA, camphorquinone (CQ) and ethyl 4-(dimethylamino)benzoat e (EDAB)	10 and 20	Dental adhesives	∼1.1	[195]
Sr-doped BG	poly(vinyl phosphonic-co-acrylic acid)	2:1 glass to polymer weight ratio	Bone cement	2.2	[196]
Sr-doped BG	PMMA	20–40	Injectable bone cement	1–2.25	[197]
ZrO_2_ and BaSO_4_	PMMA	10	Bone cement	Contrast reported	[198]
Sr-dopes HAp	PMMA	20	Injectable bone Cement	Qualitatively studied	[199]

Although many similar compositions of materials in Table 3 are currently in the market and perform very well, adding radiopacifiers may compromise biological and mechanical properties. For example, micron-sized and agglomerated particles can decrease the tensile strength of PMMA [200] and serve as the sites for fatigue crack initiation [201]. Such composites reinforced with micron-sized ceramic particles cause more bone resorption than pure PMMA, leading to increased osteolysis [202]. Radiopaque fillers in dental composite resins, adhesives, and sealers play significant roles in modifying mechanical properties such as elastic modulus, fracture toughness, strength, fatigue life, wear-resistance, and hardness. Their content, size, distribution, shape, porosity, and surface properties are crucial to achieving good packing and enhanced filler–matrix interactions to improve mechanical properties and radiopacity [203]. 

Nowadays, the harnessing of nanotechnology and the chemistry-based synthesis of materials at molecular scales are recommended as solutions for overcoming several drawbacks related to conventional polymer–ceramic composites. For example, nano-fillers/opacifiers can contribute significantly to increasing materials’ homogeneity and degradation, improving the modulus of elasticity and strength and, if needed, delivering drugs or therapeutic ions. On the other hand, inorganic–organic hybrids are relatively new materials and show low polymerization shrinkage, improved wear resistance, and biocompatibility. They are synthesized through sol-gel processing, in which a polymeric molecular precursor as a starting material is combined with metallic oxide frameworks during hydrolysis and condensation at low temperatures [122,200,204,205]. Some recent examples of such radiopaque restorative materials are provided here. 

Salarian et al. [206] pursued a new approach to preparing radiopaque and angiogenic poly(propylene fumarate) (PPF) bone cements by incorporating Sr-doped TiO_2_ nanowires and ginsenoside Rg1 suitable for treating osteonecrosis. The Sr-doped TiO_2_-nanowires had a high aspect ratio, showing a new phase, SrTiO_3_. Maleic anhydride was used to functionalize PPF and produce terminal carboxyl groups, which had strong interfacial adhesion to the nanowires. The materials presented a radiopacity of 0.3 mmAl, which was very comparable to TiO_2_-, HAp-, and BaSO_4_-added bone cements (radiopacities of 0.2, 0.15, and 0.36 mmAl, respectively). This technique also showed the excellent release of ginsenoside Rg1 in vitro, which is conducive to the cementation of necrotic bone.

Bakina et al. [161] have tried to improve the radiopacity and mechanical properties of PLA, which is known for its high biocompatibility and propitious elastic modulus, similar to that of human bone. They added magnetic Fe(core)–Fe_3_O_4_(shell) nanoparticles of 68 nm into PLA to obtain 3D-printed Fe-Fe_3_O_4_–PLA nanocomposites. The investigation suggested that 10 wt.% of Fe-Fe_3_O_4_ improves the strength of the composite and prevents its fracture along with the added value of customization (i.e., printing the anatomy of bone defects). The addition of Fe-Fe_3_O_4_ nanoparticles significantly increased the radiopacity and stimulated the growth of 3T3 fibroblast cells. The material was recommended for the 3D printing of bone scaffolds and screws. Another core–shell-structured filler was developed by Sun et al. [207] to improve the interfacial interaction between X-ray radiopaque ZrO_2_ fillers and polymer resin in dental composites. The SiO_2_ shell over ZrO_2_ microspheres was beneficial for decreasing the shrinkage (<0.1%) of the dental composite resin that exhibited a significantly enhanced radiopacity—higher than tooth enamel. 

## 7. Nanostructured Bioceramics

Intrinsically radiopaque nano-bioceramics are extensively researched as theranostic biomaterials. In this application, they can eliminate the need for two distinct biomaterials that serve cancer therapy and diagnostics/imaging. Modern theranostic bioceramics enable simultaneous diagnostic imaging, drug delivery, or other adjuvant treatments [208]. The nano-bioceramics employed for such applications include superparamagnetic iron oxide nanoparticles (SPIONs), carbon nanotubes (CNTs), and quantum dots (QDs), among others [209,210,211]. The therapeutic methods in nano-theranostics are chemotherapy, thermal ablation, photoablation, radiation therapy, and magnetic hyperthermia. 

Magnetic bioceramics are widely studied for several applications, whereas their eligibility for theranostic applications has been proved just recently. Enormous research interest in bioceramics for magnetic hyperthermia has been reported in the past decade with respect to bypassing the drawbacks of conventional cancer treatments. Designing multifunctional materials for delivering therapeutic drugs together with providing diagnostic potentials and additional therapies (e.g., magnetic hyperthermia, photothermal therapy, and radiotherapy) is at the center of current attention. For example, Sneha et al. [212] have recently developed a magnetic nanocomposite containing maghemite (γ-Fe_2_O_3_), SiO_2_, and HAp nanoparticles for a combined osteosarcoma treatment. They reported an effective hyperthermia potential, biomineralization ability, intrinsic radiopacity, and sustained doxorubicin (DOX)-release potential. The 1 mm thick magnetic nanocomposite demonstrated a contrast enhancement of 154.5% in the digital X-ray and had a HU value of 3154 analyzed by micro-CT. These interesting nano-bioceramics were added into natural rubber latex for an enhanced hyperthermia potential, better DOX loading, and sustained delivery. Although the X-ray attenuation of this nanocomposite decreased slightly due to shape adjustment, it exhibited sufficient radiopacity for diagnostic purposes, with a contrast enhancement of 121.5% and an HU value of 1353 HU [213]. 

**Figure 13:**
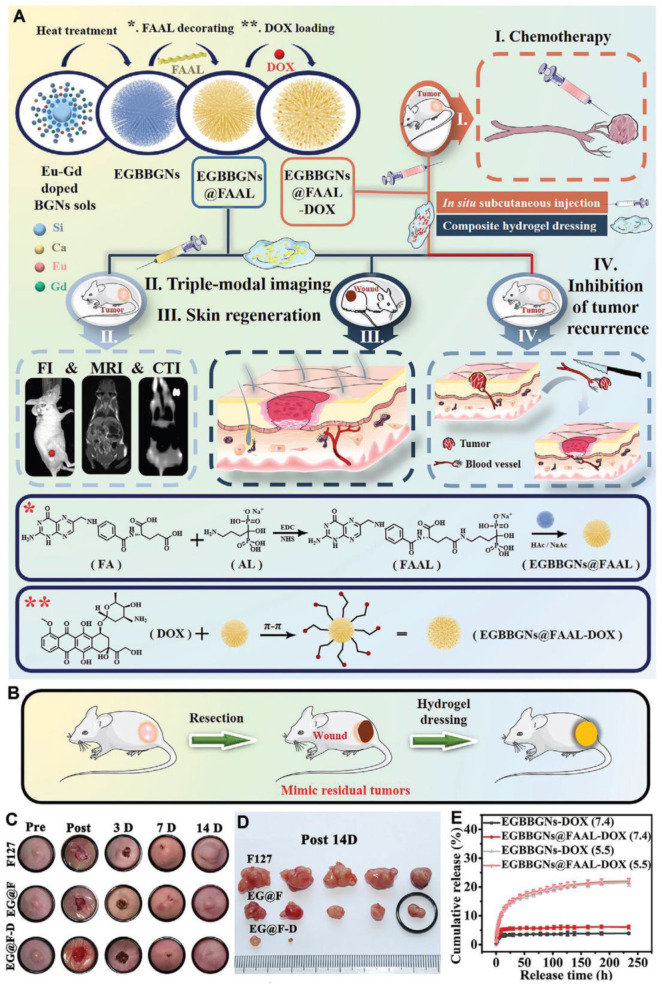
Multifunctional pH-sensitive Eu-Gd-doped MBG for skin cancer therapy and regeneration. (**A**) Schematic illustration of synthesis, decoration with folate-alendronate (FAAL), and DOX loading of the mesoporous branched bioactive glass nanoparticles (EGBBGNs). The multifunctionality of EGBBGNs including imaging, melanoma therapy, and tissue regeneration. The interactions between the nanoparticles, surface modifiers, and DOX. (**B**) Schematic of the inhibition of tumor recurrence. (**C**) Photographs related to the wounds treated with different samples as follows: EGBBGNs-FAAL (EG@F), EGBBGNs-FAAL-DOX (EG@F-D), and F127 up to 14 days. (**D**) The images related to the removed tumors of various samples. (**E**) The release profiles of different samples in physiological and acidic media. (Reproduced from [215] with permission from Elsevier.)

MBGs have also recently emerged with distinguished capabilities in cancer imaging and therapy [214]. For example, recently, a theranostic multifunctional radiopaque Eu-Gd-doped MBG decorated with alendronate and folate, loaded with DOX, was thoroughly studied for skin cancer therapy, imaging, and regeneration. An ultrahigh amount of DOX (600 mg g^−1^) could be loaded in the nano-MBG, which presented a burst release up to 24 h followed by a sustained release up to 120 h. An increased release at pH = 5.5 up to 24 h evidenced the pH-sensitive nature of the MBG [215]. The synthesis, multifunctional nature, drug release profiles, and in vivo studies on this MBG are shown in Figure 13.

Further proof of the outstanding versatility of MBGs for the development of multifunctional implantable systems was provided in a patent [216] proposing a set of injectable cements containing radiopaque Zr-doped MBG particles. The same authors demonstrated that the addition of zirconia improves the visualization of the glass under radiographic imaging without significantly altering the mesoporous texture and, hence, the good bioactive properties that can be maintained after incorporation in an injectable matrix (e.g., calcium sulfate) [146]. The in vitro biomineralization and injectability of these MBG-containing cements in ex-vivo sheep vertebrae were also demonstrated [217]. The setting time (8–20 min) and radiopacity were comparable with those of commercial PMMA cements used as references while the compressive strength was higher than that of healthy cancellous bone [218].

## 8. Conclusion and Perspective

The existing literature clearly attests to the suitability of a number of bioceramics as biocompatible radiopaque substances to be used alone (e.g., in the form of particles or porous scaffolds) or in combination with other phases (e.g., embedded in polymer-based composites) to improve the visualization of implants under radiographic imaging. The radiopacity of bioceramics can be typically potentiated by adding heavy elements or oxides into the basic material’s formulation. However, heavy metals can elicit cytotoxicity and genotoxicity in the human body depending on multiple factors, including the element concentration and the specific ceramic material in which they are incorporated, as different bioceramics have different chemical stabilities and dissolution rates upon contact with biological fluids. In this regard, it is instructive to mention the case of Bi- and Ba-doped dental bioceramics: although there are several commercial products based on these formulations that have been used in clinics for many years (see Table 1), some concerns about barium and bismuth toxicity exist and still are a matter of debate, suggesting the need for further studies, especially with respect to the long-term in vivo effects. 

It is also worth highlighting that the metallic ions improving radiopacity (diagnostic purpose) can also be considered for other therapeutic extra-functionalities, thereby facilitating the development of theranostic systems by using a single ion. For example, Zr was shown to improve the bone-regenerative properties of MBGs [219,220], and the same ion was also utilized in another study to enhance radiographic visualization [146]. However, to the best of our knowledge, both effects have not yet been reported in one unified study that addresses the theranostic potential of Zr-doped MBGs. The same concepts can be applied to other metallic elements, such as Fe: Miola et al. [221] first incorporated Fe and Ag into bioactive silicate glass-ceramics to obtain materials with triple functionalities, i.e., magnetic hyperthermia to combat cancer (due to the presence of magnetite), bone-regenerative ability, and antibacterial effects (due to the release of Ag^+^ ions), and then embedded these inorganic particles within a PMMA matrix to obtain an injectable, multifunctional bone cement. Such composite materials are also expected to be radiopaque due to the presence of iron oxide, although this specific property was not reported in that study. Therefore, reviewing the literature and rethinking the properties that can be imparted by metallic elements to bioactive ceramics and glasses can be useful for finding new possibilities—e.g., radiopacity—that were not initially considered for a single element.

The development of theranostic systems is particularly appealing with respect to MBGs, which can act not only as matrices for the incorporation of therapeutic and/or radiopacifying dopants but also serve as vehicles for the controlled release of drugs and growth factors eliciting osteogenic, angiogenic, anticancer, and antibacterial activities [222,223]. Indeed, as MBGs have an inherent porous texture, the impact of pore characteristics (e.g., total volume, shape, and interconnectivity) on radiopacity deserves to be elucidated.

Alternatively, the development of composites and cements with multiphase ceramic inclusions (e.g., the addition of heavy metal-doped BGs, MBGs, and HAp) for more selectively and finely improving a given property (e.g., radiopacity, bioactivity, and drug delivery) can also be a valuable option [218].

Apart from silicate-, phosphate-, sulfate-, and, in general, oxide-based ceramics, other types of advanced biomaterials such as carbon nanotubes (CNTs) [224] or metal-organic frameworks (MOFs) [225] could be functionalized with radiopaque heavy elements to then be embedded in polymeric matrices or used as contrast agents.

Examining composite materials, the implementation of theoretical and experimental studies to model and simulate the packing ability and distribution of the filler phase throughout the volume of the matrix, as well as the optimal concentration and morphology of radiopaque ceramic inclusions, will be highly beneficial in the context of radiopacity to obtain an adequate radio-visualization effect.

Numerical studies assisted by machine learning could also be carried out to model radiopacity, as already performed, for example, for other properties of bioactive glasses (e.g., dissolution kinetics, mineralization ability, etc.) [226].

Finally, testing and comparing the radiopacity of new ceramic and glass compositions should require some refinement and standardization for clinical applications. The attenuative effect provided by the material should be investigated by applying the X-ray tube voltages used clinically along with water phantoms mimicking the effect of a patient’s soft tissue. In this regard, Kjellson et al. [198] recommended testing bone cements for hip and knee prostheses under a voltage of 70–80 kV along with the interposition of around 10 cm of water to obtain reliable results in terms of radiographic contrast.

## Figures and Tables

**Table 1:** Some commercial radiopaque bioceramics used in dentistry.

Material	Composition	Radiopacifying	Company	Ref.
ProRoot MTA	Portland cement 75% Calcium sulfate dihydrate 5% Bismuth oxide 20%	Bismuth oxide	Dentsply Tulsa Dental, Tulsa, OK, USA	[48]
RetroMTA	Calcium Carbonate 60–80% Silicon dioxide 5–15% Aluminum oxide 5–10% Calcium zirconia complex 20–30%	Zirconia complex	BioMTA, Seoul, Korea	[48]
BioMTA	Powder: Calcium carbonate, silicon dioxide, aluminum oxide, and calcium zirconia complex. Liquid: Distilled water	Zirconia complex	Intradent, Belém, PA, Brazil	[49]
MTA Angelus	Powder: silicon dioxide, potassium oxide, aluminum oxide, sodium oxide, ferric oxide, sulfur trioxide, calcium oxide, bismuth oxide, magnesium oxide. Insoluble residues of calcium oxide, potassium sulfate, sodium sulfate, and crystalline silica. Liquid: distilled water.	Bismuth oxide	Angelus, Londrina, PR, Brazil	[49]
Endosequence BC Sealer	Zirconium oxide, calcium silicates, calcium phosphate monobasic, calcium hydroxide, filler, and thickening agents	Zirconium oxide	Brasseler, Savannah, GA, USA	[50]
Total Fill BC sealer	Zirconium oxide (35–45%), tricalcium silicate (20–35%), dicalcium silicate (7–15%), and calcium hydroxide (1–4%)	Zirconium oxide	FKG Dentaire, Switzerland	[51]
AH Plus	Epoxy paste: diepoxy, calcium tungstate, zirconium oxide, aerosol, and dye. Amine paste: 1-adamantane amine, N.N’dibenzy l-5 oxanonandiamine-1,9, TCD-diamine, calcium tungstate, zirconium oxide, aerosol, and silicon oil.	Zirconium oxide	Dentsply De Trey Gmbh, Konstanz, Germany	[50]
Ceramir^®^ Bioceramic Implant Cement QuikCap	Polyacrylic acid (<10%) Strontium fluoride (<5%) Tartaric acid (<5%)	Strontium fluoride	Doxa Dental AB, Sweden	[24]
Surefil one	Aluminum-phosphor-strontium-sodium-fluoro-silicate glass, water, highly dispersed silicon dioxide, acrylic acid, polycarboxylic acid (MOPOS), ytterbium fluoride, bifunctional acrylate (BADEP), self-cure initiator, iron oxide pigments, barium sulfate pigment, manganese pigment, camphorquinone, stabilizer	Glass Ytterbium fluoride Barium sulfate	Dentsply Sirona, Konstanz, Germany	[52]
Fuji IX GP Fast	Aluminofluorosilicate glass, polyacrylic acid, distilled water, poly carboxylic acid	Glass	GC Corporation, Tokyo, Japan	[53]
Ketac™ Molar Quick Aplicap™	Al-Ca-La fluorosilicate glass, 5% copolymer acid (acrylic and maleic acid), Polyalkenoic acid, tartaric acid, water	Glass	3M ESPE, Deutschland, Germany	[54]

**Table 2:** The radiopacity values of different ceramic materials incorporated with various radiopacifying agents. MTA stands for mineral trioxide aggregate.

Radiopacifying Agents (Element/Compounds)		Proportion Used	Host Bioceramic	Radiopacity	Ref.
Bi	Bi_2_O_3_	20%	Calcium silicate cement	5.78 ± 0.5 mmAl	[83]
	Bi-doped	0.1 mol	Baghdadite	Increased by 33%	[85]
	Bi_2_O_3_	15–25%	MTA cement	4.3 to 6.0 mmAl	[91]
	Bi_2_(Al_2_O_4_)_3_	9–15%	Calcium phosphate cement	1.86 to 2.88 mmAl	[92]
Zr	ZrO_2_	30%	Calcium silicate cement	5.94 ± 0.9 mmAl	[83]
		20–40%	Calcium phosphate cement	1.5 to 2.5 mmAl	[93]
	ZrO_2_ short fiber	2–8%	Calcium phosphate cement	Increased by 12%	[94]
	Bi_1.8_Zr_0.2_O_3.1_	0.2 mol	MTA	5.57 ± 0.28 mmAl	[95]
Sr	Sr-doped	8.2–24.6%	Magnesium phosphate scaffolds	1.2 to 2.0 mmAl	[96]
		1.10–2.21%	Tricalcium phosphate cement	2.0 to 3.0 mmAl	[97]
		10%	Tricalcium silicate cement	Increased by 25%	[85]
Ba	BaSO_4_	20%	Portland cement	2.35 ± 0.08 mmAl	[80]
		25%		3.5 mmAl	[98]
W	CaWO_4_	30%	Calcium silicate cement	5.67 ± 0.5 mmAl	[83]
		10–30%	Calcium silicate particles	3.24 to 3.85 mmAl	[87]
Fe	Fe_2_O_3_	20–60%	HAp	Increased up to 38%	[86]
Yb	Yb_2_O_3_	30%	Calcium silicate cement	5.02 ± 0.43 mmAl	[89]

## Data Availability

Not applicable.
